# Supplementary material for: Enhanced multi-colour gating for the generation of high-power isolated attosecond pulses
Source: Sci Rep. 2015 May 22;5:10084. doi: 10.1038/srep10084 (PMC4441196; doi:10.1038/srep10084)
Supplement: Supplementary Information [file srep10084-s1.pdf]

Supplementary information to "Enhanced multi-colour gating for the generation of high-power isolated attosecond pulses"

S. Haessler,[1,2] T. Balčiunas,[2] G. Fan,[2] L. E. Chipperfield,[3] and A. Baltuška[2]

[1] *Laboratoire d'Optique Appliqueé, ENSTA-Paristech, Ecole Polytechnique, CNRS, 91761 Palaiseau Cedex, France*
[2] *Photonics Institute, Vienna University of Technology, Gußhausstraße 27/387, 1040 Vienna, Austria*
[3] *The Blackett Laboratory, Imperial College London, South Kensington Campus, London SW7 2AZ, United Kingdom*

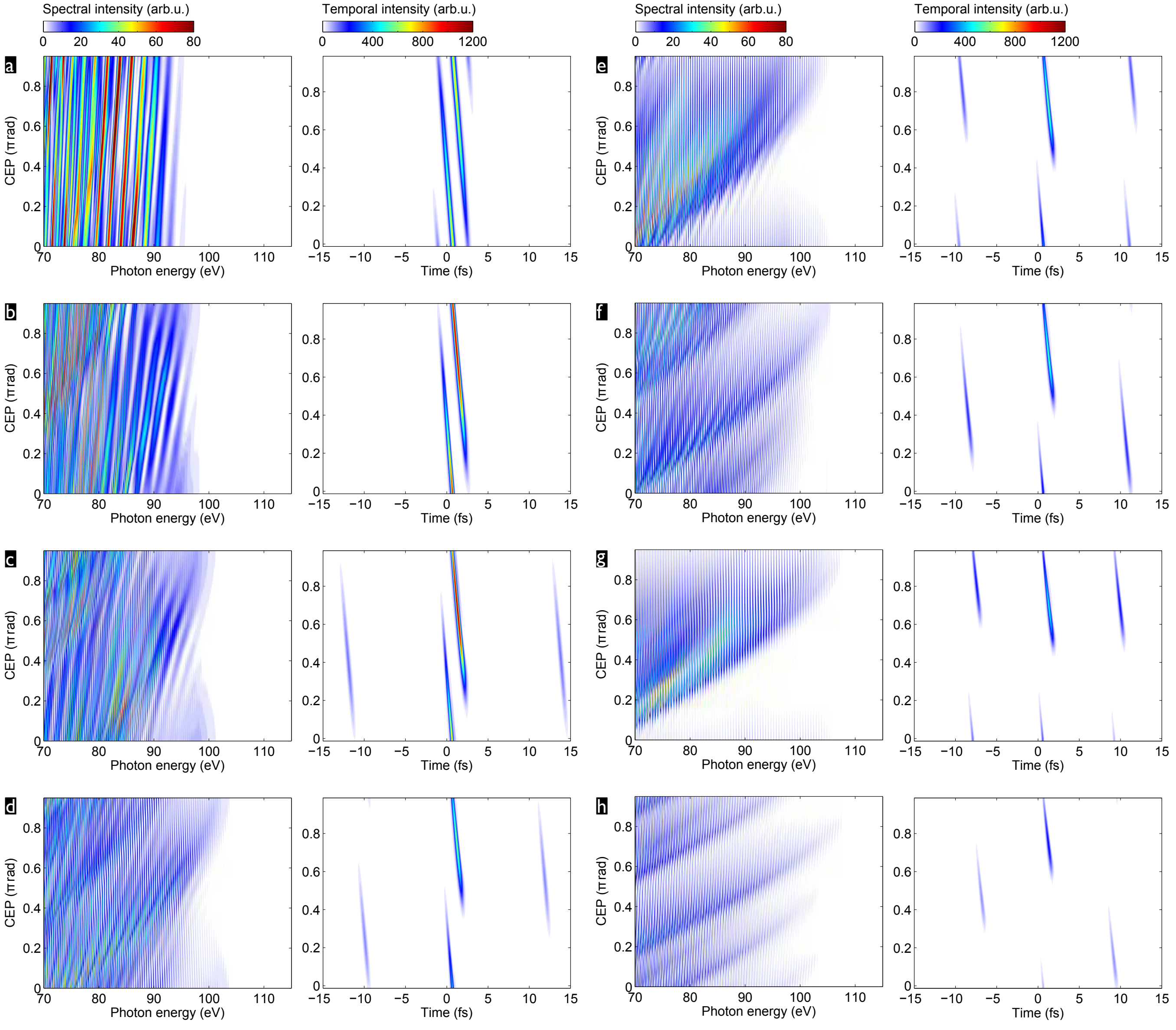

FIG. S1. *Equivalent to Fig. 3 of the main paper, but for* $R = 1/2$. CEP-dependence of the generated high harmonic spectrum (left) and attosecond pulses (right) for the combination of a 1030-nm fundamental with an auxiliary 1200-nm (a), 1300-nm (b), 1400-nm (c), 1500-nm (d), 1545-nm (e), 1600-nm (f), 1700-nm (g), or 1800-nm (h) pulse, both with a 60-fs duration. The pulses have peak intensities of $I_0 = 2/3 \times 10^{14}$ W cm^{-2} and $I_1 = 1/3 \times 10^{14}$ W cm^{-2}, respectively, i.e. $R = 1/2$. For the attosecond pulses, the cutoff region has been selected by a 15-eV wide normalized super-Gaussian filter centered at 95 eV (a-c), 100 eV (d-g) or 105 eV (h) (cp. figure S4). Outside of the shown time window, the attosecond emission is suppressed to below a 10^{-3}-fraction of the strongest attosecond pulse's peak intensity.

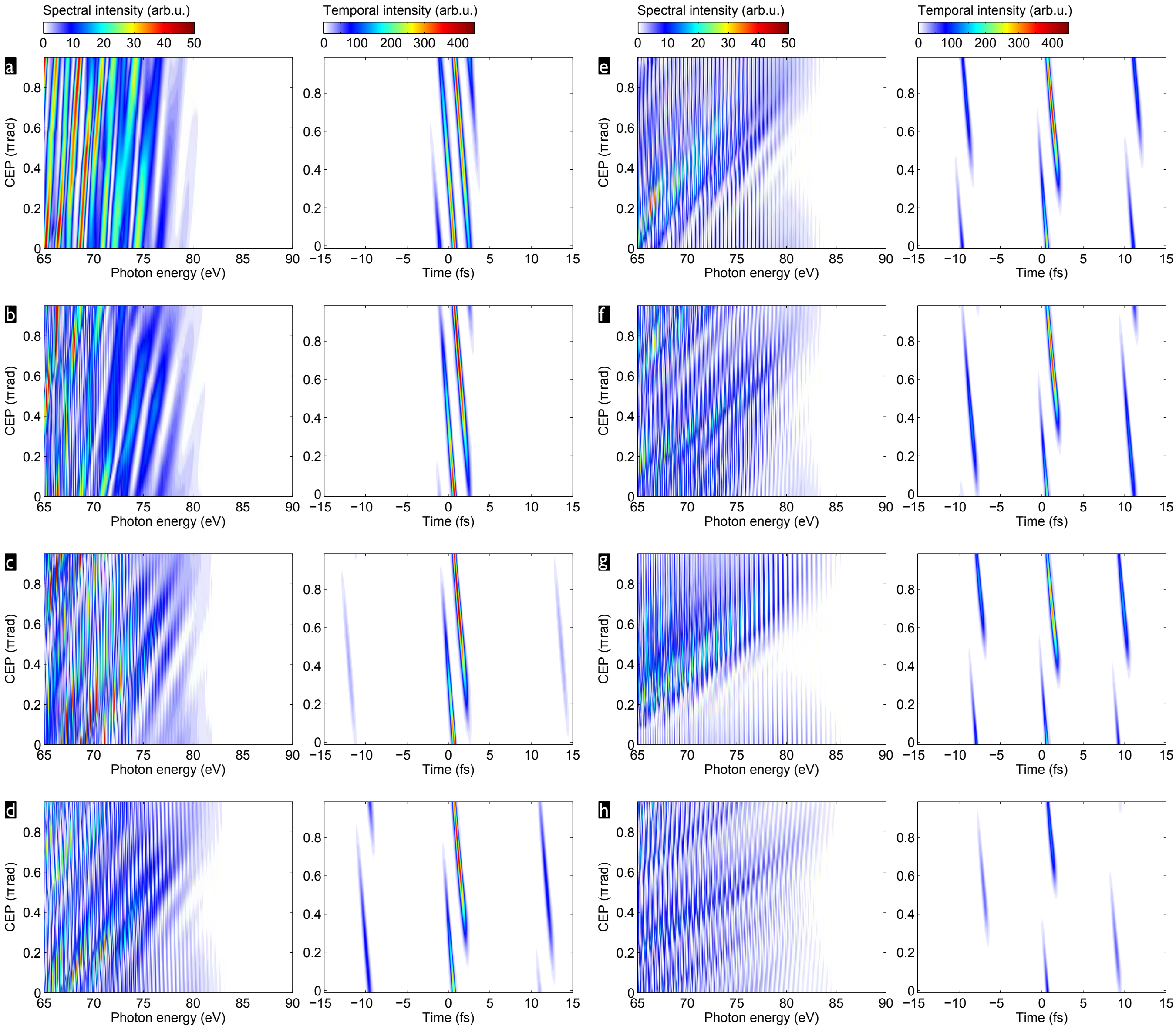

FIG. S2. *Equivalent to Fig. 3 of the main paper, but for $R = 1/9$.* CEP-dependence of the generated high harmonic spectrum (left) and attosecond pulses (right) for the combination of a 1030-nm fundamental with an auxiliary 1200-nm (a), 1300-nm (b), 1400-nm (c), 1500-nm (d), 1545-nm (e), 1600-nm (f), 1700-nm (g), or 1800-nm (h) pulse, both with a 60-fs duration. The pulses have peak intensities of $I_0 = 0.9 \times 10^{14}$ W cm^{-2} and $I_1 = 0.1 \times 10^{14}$ W cm^{-2}, respectively, *i.e.* $R = 1/9$. For the attosecond pulses, the cutoff region has been selected by a 15-eV wide normalized super-Gaussian filter centered at 80 eV (a-g) or 85 eV (h) (cp. figure S5). Outside of the shown time window, the attosecond emission is suppressed to below a 10^{-3}-fraction of the strongest attosecond pulse's peak intensity.

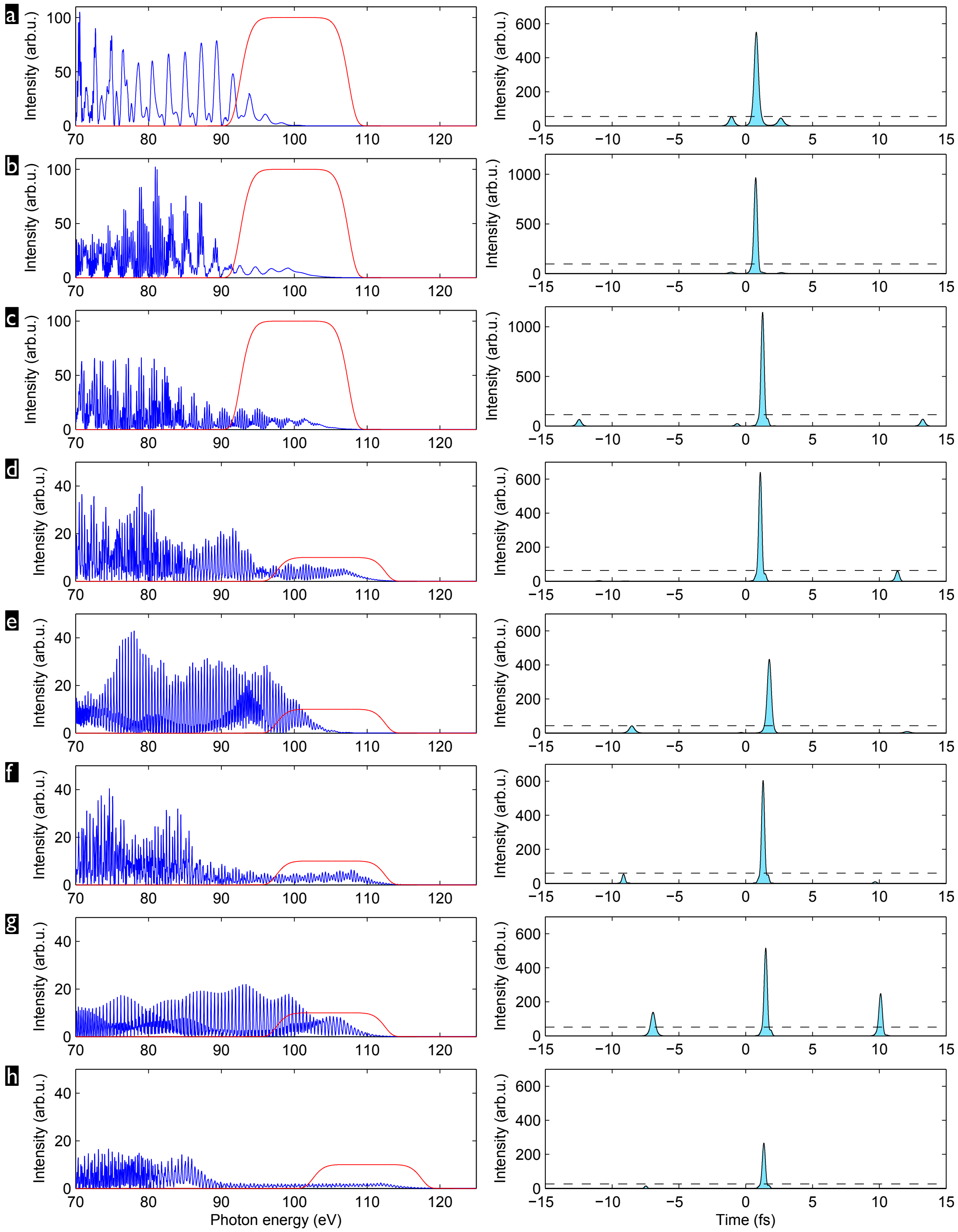

FIG. S3. High harmonic spectrum (left) and attosecond pulses (right) for selected optimal phase delays out of the scans in figure 3 of the main paper ($R = 1$): for the auxiliary 1200-nm pulse, $\varphi_{\mathrm{CEP}} = 0$ rad (a), for 1300-nm, $\varphi_{\mathrm{CEP}} = 0$ rad (b), for 1400-nm, $\varphi_{\mathrm{CEP}} = 0.7\pi$ rad (c), for 1500-nm, $\varphi_{\mathrm{CEP}} = 0.8\pi$ rad (d), for 1545-nm, $\varphi_{\mathrm{CEP}} = 0.55$ rad (e), for 1600-nm, $\varphi_{\mathrm{CEP}} = 0.7\pi$ rad (f), for 1700-nm, $\varphi_{\mathrm{CEP}} = 0.7\pi$ rad (g), for 1800-nm, $\varphi_{\mathrm{CEP}} = 0.7\pi$ rad (h). The red line superposed to the spectra shows the 15-eV wide normalized super-Gaussian filter centered at 100 eV (a-c), 105 eV (d-f) or 110 eV (g,h), which selects the cutoff region for the attosecond pulses. The dashed line marks the 10%-level of the intensity of the strongest attosecond pulse.

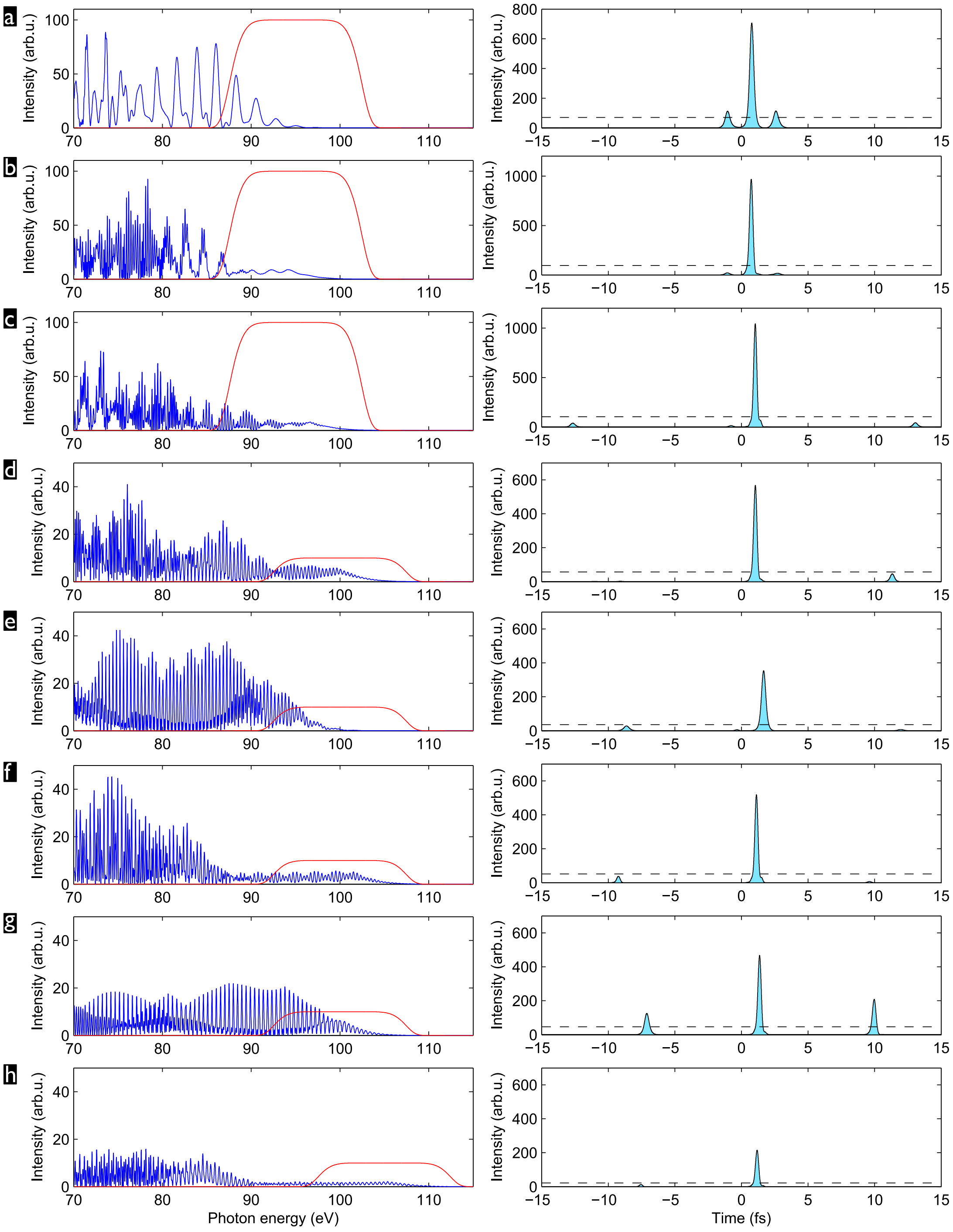

FIG. S4. *Equivalent to Fig. S3, but for* $R = 1/2$. High harmonic spectrum (left) and attosecond pulses (right) for selected optimal phase delays out of the scans in figure S1: for the auxiliary 1200-nm pulse, $\varphi_{\mathrm{CEP}} = 0\,\mathrm{rad}$ (a), for 1300-nm, $\varphi_{\mathrm{CEP}} = 0\,\mathrm{rad}$ (b), for 1400-nm, $\varphi_{\mathrm{CEP}} = 0.8\pi$ rad (c), for 1500-nm, $\varphi_{\mathrm{CEP}} = 0.8\pi$ rad (d), for 1545-nm, $\varphi_{\mathrm{CEP}} = 0.55\pi$ rad (e), for 1600-nm, $\varphi_{\mathrm{CEP}} = 0.8\pi$ rad (f), for 1700-nm, $\varphi_{\mathrm{CEP}} = 0.7\pi$ rad (g), for 1800-nm, $\varphi_{\mathrm{CEP}} = 0.8\pi$ rad (h). The red line superposed to the spectra shows the 15-eV wide normalized super-Gaussian filter centered at 95 eV (a-c), 100 eV (d-g) or 105 eV (h), which selects the cutoff region for the attosecond pulses. The dashed line marks the 10%-level of the intensity of the strongest attosecond pulse.

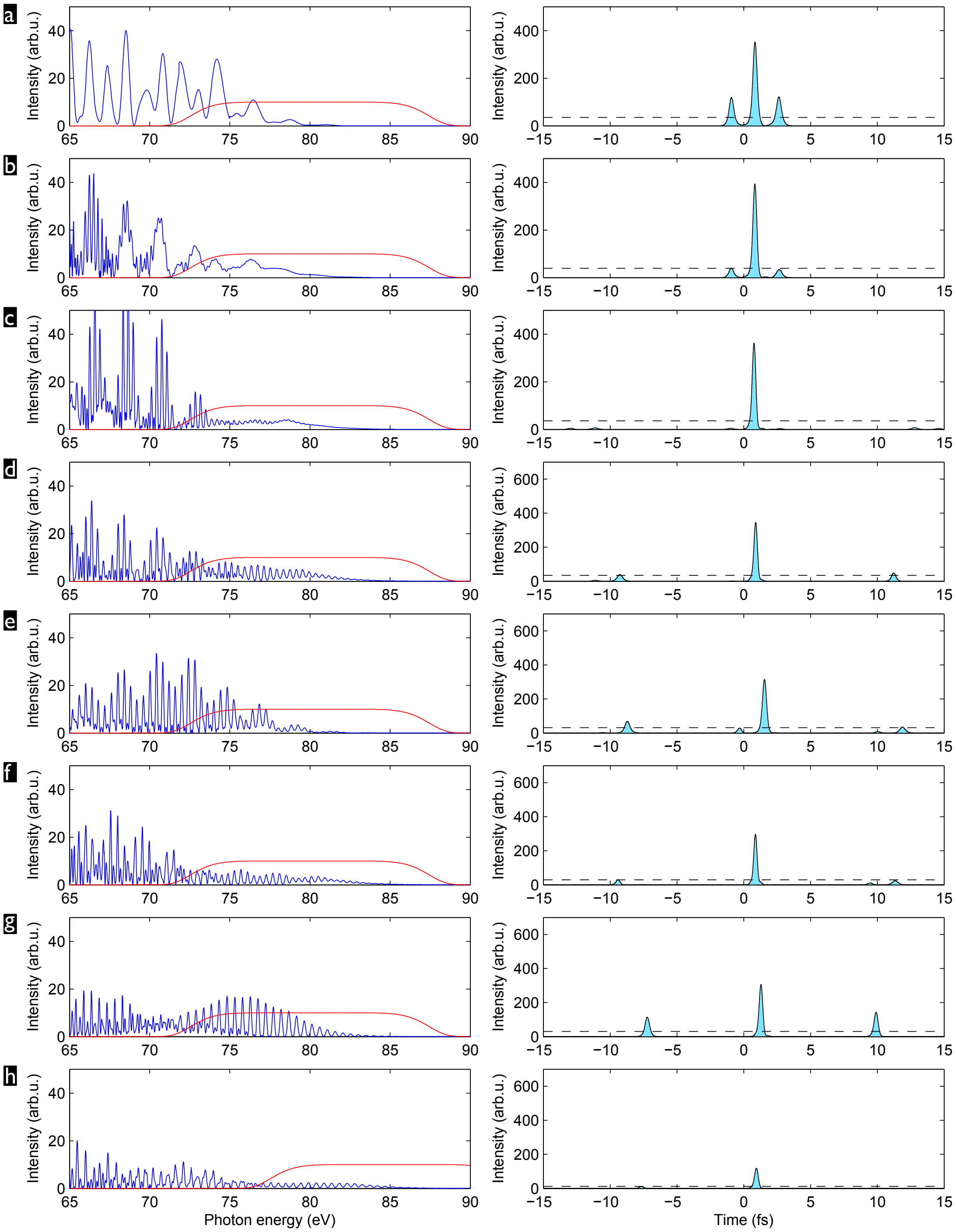

FIG. S5. *Equivalent to Fig. S3, but for* $R = 1/9$. High harmonic spectrum (left) and attosecond pulses (right) for selected optimal phase delays out of the scans in figure S2: for the auxiliary 1200-nm pulse, $\varphi_{\rm CEP} = 0.9\pi$ rad (a), for 1300-nm, $\varphi_{\rm CEP} = 0.9\pi$ rad (b), for 1400-nm, $\varphi_{\rm CEP} = 0.95\pi$ rad (c), for 1500-nm, $\varphi_{\rm CEP} = 0.9\pi$ rad (d), for 1545-nm, $\varphi_{\rm CEP} = 0.55\pi$ rad (e), for 1600-nm, $\varphi_{\rm CEP} = 0.9\pi$ rad (f), for 1700-nm, $\varphi_{\rm CEP} = 0.7\pi$ rad (g), for 1800-nm, $\varphi_{\rm CEP} = 0.9\pi$ rad (h). The red line superposed to the spectra shows the 15-eV wide normalized super-Gaussian filter centered at 80 eV (a-g) or 85 eV (h), which selects the cutoff region for the attosecond pulses. The dashed line marks the 10%-level of the intensity of the strongest attosecond pulse.

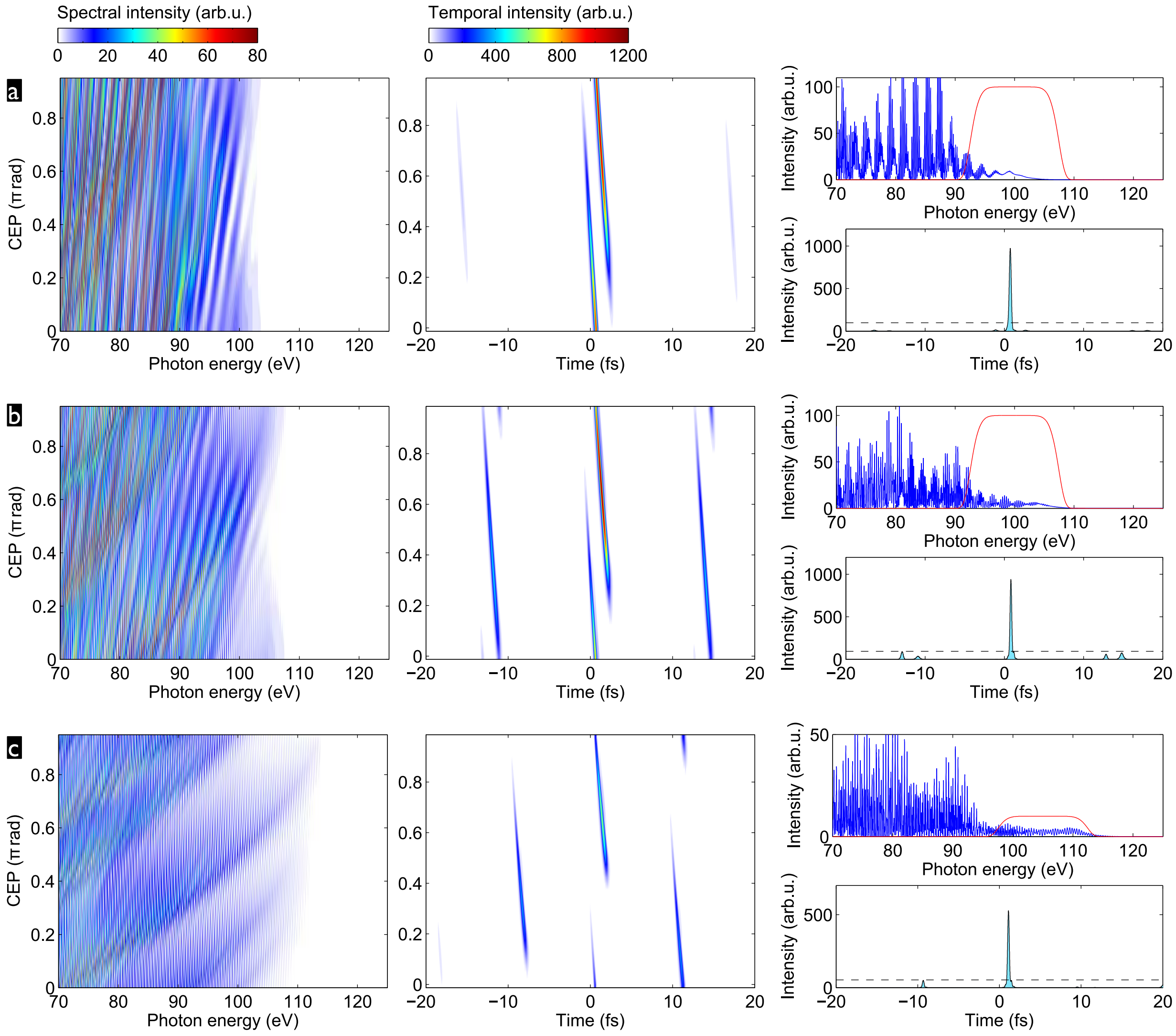

FIG. S6. CEP-dependence of the generated high harmonic spectrum (left) and attosecond pulses (middle) for the combination of a 180-fs, 1030-nm fundamental with an auxiliary *non-integer-N* 1300-nm (a), 1400-nm (b), or 1600-nm (c) pulse of 60 fs duration. The pulses have peak intensities of $I_0 = 0.5 \times 10^{14}\ \mathrm{W\,cm^{-2}}$ and $I_1 = 0.5 \times 10^{14}\ \mathrm{W\,cm^{-2}}$, respectively, *i.e.* $R = 1$. For the attosecond pulses, the cutoff region has been selected by a 15-eV wide normalized super-Gaussian filter centered at 100 eV (a,b) or 105 eV (c), shown by the red line superposed to the spectra on the right. Spectra and attosecond pulses for the selected optimal $\varphi_{\mathrm{CEP}} = 0\,\mathrm{rad}$ (a), $\varphi_{\mathrm{CEP}} = 0.9\pi\,\mathrm{rad}$ (b) and $\varphi_{\mathrm{CEP}} = 0.8\pi\,\mathrm{rad}$ (c) (right). The dashed line marks the 10%-level of the intensity of the strongest attosecond pulse. Outside of the shown time window, the attosecond emission is suppressed to below a 10^{-4}-fraction of the strongest attosecond pulse's peak intensity.

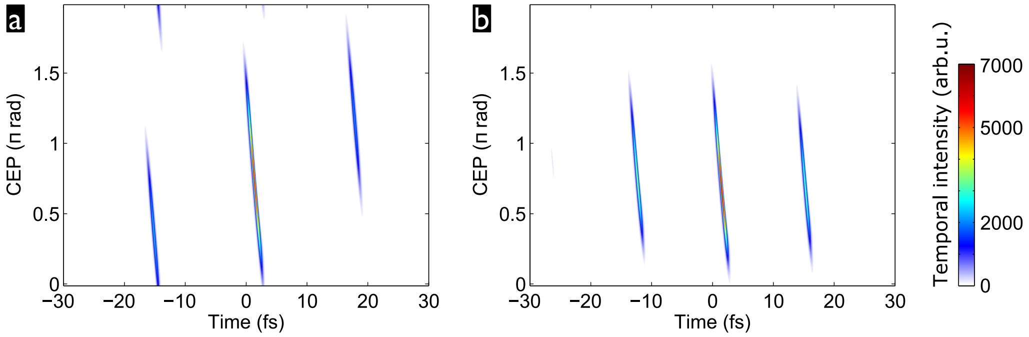

FIG. S7. CEP-dependence of the attosecond pulses generated by a three-colour field composed of a 180-fs, 1030-nm fundamental pulse, its 180-fs, 515-nm second harmonic with $\varphi_2 = 0.1\pi$, and an auxiliary pulse with 100-fs duration and $\lambda_1 = 1300$ nm (*non-integer* $N = 8.6$) (a), $\lambda_1 = 1370$ nm (*odd* $N = 7$) (b). The pulses have peak intensities of $I_0 = 0.48 \times 10^{14}$ W cm^{-2}, $I_2 = 0.05 \times 10^{14}$ W cm^{-2}, and $I_1 = 0.48 \times 10^{14}$ W cm^{-2}, respectively, *i.e.* $R = 1$. For the attosecond pulses, the cutoff region has been selected by a 15-eV wide normalized super-Gaussian filter centered at 100 eV. No IAP can be generated with these auxiliary wavelenegths.
